# Evolutionary restoration of fertility in an interspecies hybrid yeast, by whole-genome duplication after a failed mating-type switch

**DOI:** 10.1371/journal.pbio.2002128

**Published:** 2017-05-16

**Authors:** Raúl A. Ortiz-Merino, Nurzhan Kuanyshev, Stephanie Braun-Galleani, Kevin P. Byrne, Danilo Porro, Paola Branduardi, Kenneth H. Wolfe

**Affiliations:** 1 UCD Conway Institute, School of Medicine, University College Dublin, Dublin, Ireland; 2 Department of Biotechnology and Biosciences, University of Milano-Bicocca, Milano, Italy; University of Bath, United Kingdom of Great Britain and Northern Ireland

## Abstract

Many interspecies hybrids have been discovered in yeasts, but most of these hybrids are asexual and can replicate only mitotically. Whole-genome duplication has been proposed as a mechanism by which interspecies hybrids can regain fertility, restoring their ability to perform meiosis and sporulate. Here, we show that this process occurred naturally during the evolution of *Zygosaccharomyces parabailii*, an interspecies hybrid that was formed by mating between 2 parents that differed by 7% in genome sequence and by many interchromosomal rearrangements. Surprisingly, *Z*. *parabailii* has a full sexual cycle and is genetically haploid. It goes through mating-type switching and autodiploidization, followed by immediate sporulation. We identified the key evolutionary event that enabled *Z*. *parabailii* to regain fertility, which was breakage of 1 of the 2 homeologous copies of the mating-type (*MAT*) locus in the hybrid, resulting in a chromosomal rearrangement and irreparable damage to 1 *MAT* locus. This rearrangement was caused by HO endonuclease, which normally functions in mating-type switching. With 1 copy of *MAT* inactivated, the interspecies hybrid now behaves as a haploid. Our results provide the first demonstration that *MAT* locus damage is a naturally occurring evolutionary mechanism for whole-genome duplication and restoration of fertility to interspecies hybrids. The events that occurred in *Z*. *parabailii* strongly resemble those postulated to have caused ancient whole-genome duplication in an ancestor of *Saccharomyces cerevisiae*.

## Author summary

It has recently been proposed that the whole-genome duplication (WGD) event that occurred during evolution of an ancestor of the yeast *S*. *cerevisiae* was the result of a hybridization between 2 parental yeast species that were significantly divergent in DNA sequence, followed by a doubling of the genome content to restore the hybrid’s ability to make viable spores. However, the molecular details of how genome doubling could occur in a hybrid were unclear because most known interspecies hybrid yeasts have no sexual cycle. We show here that *Z*. *parabailii* provides an almost exact precedent for the steps proposed to have occurred during the *S*. *cerevisiae* WGD. Two divergent haploid parental species, each with 8 chromosomes, mated to form a hybrid that was initially sterile but regained fertility when 1 copy of its mating-type locus became damaged by the mating-type switching apparatus. As a result of this damage, the *Z*. *parabailii* life cycle now consists of a 16-chromosome haploid phase and a transient 32-chromosome diploid phase. Each pair of homeologous genes behaves as 2 independent Mendelian loci during meiosis.

## Introduction

A whole-genome duplication (WGD) occurred more than 100 million years ago in the common ancestor of 6 yeast genera in the ascomycete family Saccharomycetaceae, including *Saccharomyces* [1, 2]. Recent phylogenomic analysis has shown that the WGD was an allopolyploidization—that is, a hybridization between 2 different parental lineages [3]. One of these parental lineages was most closely related to a clade containing *Zygosaccharomyces* and *Torulaspora* (ZT), whereas the other was closer to a clade containing *Kluyveromyces*, *Lachancea*, and *Eremothecium* (KLE). The ZT and KLE clades are the 2 major groups of non-WGD species in family Saccharomycetaceae. The WGD had a profound effect on the genome, proteome, physiology, and cell biology of the yeasts that are descended from it, but the genomes of these yeasts have changed substantially in the time since the WGD occurred, with extensive chromosomal rearrangement, deletion of duplicate gene copies, and sequence divergence between ohnologs (pairs of paralogous genes produced by the WGD). These changes have made it difficult to ascertain the molecular details of how the WGD occurred. Ancient hybridizations are rare in fungi (or at least difficult to detect [4]), but numerous relatively recent hybridizations have been identified using genomics, particularly in the ascomycete genera *Saccharomyces* [5, 6], *Zygosaccharomyces* [7–9], *Candida* [10–12], and *Millerozyma* [13].

Marcet-Houben and Gabaldón [3] proposed 2 alternative hypotheses for the mechanism of interspecies hybridization that led to the ancient WGD in the *Saccharomyces* lineage. Hypothesis A was hybridization between diploid cells from the 2 parental species, perhaps by cell fusion. Hypothesis B was mating between haploid cells from the 2 parental species to produce an interspecies hybrid zygote, followed by genome doubling. Under both hypotheses, the product is a cell with 2 identical copies of each parental chromosome. These identical copies should be able to pair during meiosis, leading to viable spores. While there are no known examples of natural yeast hybrid species formed by diploid–diploid fusion (hypothesis A), 3 examples have been discovered in which hybrid species were apparently formed simply by mating between haploids of opposite mating types from different species (hypothesis B). These are *Candida metapsilosis* [11], *C*. *orthopsilosis* [10, 12], and *Zygosaccharomyces* strain ATCC42981 [8, 14]. These interspecies hybridizations occurred by mating between parents with 4%–15% nucleotide sequence divergence between their genomes. However, none of these 3 hybrids can sporulate, which could be either because the homeologous chromosomes from the 2 parents are too divergent in sequence to pair up during meiosis or because pairing occurs but evolutionary rearrangements (such as translocations) between the parental karyotypes result in DNA duplications or deficiencies after meiosis [15–18]. None of these 3 hybrids has undergone the genome-doubling step envisaged in hypothesis B.

Several groups [3, 18–20] have proposed that genome doubling could occur quite simply by means of damage to 1 copy of the *MAT* locus in the interspecies hybrid, which could cause the hybrid cell to behave as a haploid, switch mating type, and hence autodiploidize. This proposal mimics laboratory experiments carried out by Greig et al. [21] in which hybrids between different species of *Saccharomyces* were constructed by mating. The hybrids were unable to segregate chromosomes properly and were sterile, but when 1 allele of the *MAT* locus was deleted, they spontaneously autodiploidized by mating-type switching and were then able to complete meiosis and produce spores with high viability. Each spore contained a full set of chromosomes from both parental species [21]. While genome doubling via *MAT* locus damage is an attractive hypothesis consistent with hypothesis B above [3], no examples of it occurring in nature have been described. We show here that *Z*. *parabailii* has gone through this process.

There are 12 formally described species in the genus *Zygosaccharomyces* [22]. The most studied of these is *Z*. *rouxii*, originally found in soy sauce and miso paste [23, 24]. Others include *Z*. *mellis*, frequently found in honey [25], and *Z*. *sapae* from balsamic vinegar [26, 27]. Species in the *Z*. *bailii* sensu lato clade (*Z*. *bailii*, *Z*. *parabailii*, and *Z*. *pseudobailii*; [28]) are of economic importance because they are exceptionally resistant to osmotic stress and low pH. Their resistance to the weak organic acids commonly used as food preservatives makes them the most frequent spoilage agent of packaged foods with high sugar content, such as fruit juices and jams, or with low pH, such as mayonnaise [29–33]. These same characteristics make *Zygosaccharomyces* relevant to biotechnology since high stress tolerance and rapid growth are often desirable traits in microorganisms to be used as cell factories. The strain we analyze here, *Z*. *parabailii* ATCC60483, has previously been used for production of vitamin C [34], lactic acid [35], and heterologous proteins [36].

Despite the diversity of the genus, genome sequences have been published for only 2 nonhybrid species of *Zygosaccharomyces*: the type strains of *Z*. *rouxii* (CBS732^T^; [37]) and *Z*. *bailii* (CLIB213^T^; [38]). The genus also includes many interspecies hybrids with approximately twice the DNA content of pure species (20 Mb instead of 10 Mb; [7, 8, 14, 39]). Mira et al. [39] sequenced the genome of *Zygosaccharomyces* strain ISA1307 and found that it is a hybrid between *Z*. *bailii* and an unidentified *Zygosaccharomyces* species. In 2013, Suh et al. [28] proposed that some strains that were historically classified as *Z*. *bailii* should be reclassified as 2 new species, *Z*. *parabailii* and *Z*. *pseudobailii*, based on phylogenetic analysis of a small number of genes. The sequences of the *RPB1* and *RPB2* genes that they obtained from *Z*. *parabailii* and *Z*. *pseudobailii* contained multiple ambiguous bases, consistent with a hybrid nature [39]. In the current study, we sequenced the genome of a second hybrid strain, ATCC60483. We show that ATCC60483 and ISA1307 are both *Z*. *parabailii* and are both descended from the same interspecies hybridization event. By sequencing ATCC60483 using Pacific Biosciences (PacBio) technology, we obtained near-complete sequences of every *Z*. *parabailii* chromosome, which enabled us to study aspects of chromosome evolution in this species that were not evident from the Illumina assembly of ISA1307 [39].

## Results

### *Z*. *parabailii* ATCC60483 genome assembly by PacBio

We first tried to sequence the *Z*. *parabailii* genome using Illumina technology, but even with high coverage, we were unable to obtain long contigs. The data indicated that the genome was a hybrid, so instead we switched to PacBio technology, which generates long sequence reads (6 kb on average in our data). Our initial assembly had 22 nuclear scaffolds, which we refined into 16 complete chromosome sequences with a cumulative size of 20.8 Mb by manually identifying overlaps between the ends of scaffolds and by tracking centromere and telomere locations. We annotated genes using the Yeast Genome Annotation Pipeline (YGAP), assisted by RNA sequencing (RNA-Seq) data to identify introns. The nuclear genome has 10,087 protein-coding genes, almost twice as many as *Z*. *bailii* CLIB213^T^ (Table 1).

**Table 1:** Comparison of *Z*. *bailii* and *Z*. *parabailii* genome assemblies.

Strain	Species	Genome size (Mb)	Scaffolds	Scaffold N50 (Mb)	Reference	tRNA genes^a^	Protein-coding genes
CLIB213^T^	*Z*. *bailii*	10.2	27	0.9	Galeote et al. [38]	161	5,084
ISA1307	*Z*. *parabailii*	21.2	154	0.2	Mira et al. [39]	513	9,925
ATCC60483	*Z*. *parabailii*	20.8	16	1.3	This study	499	10,087

^a^ We predicted the tRNA gene content of each genome assembly using tRNAscan-SE [40].

Most of the chromosome sequences extend into telomeric repeats at the ends. The consensus sequence of the telomeres is tgtgggtgggg, which matches exactly the sequence of the template region of the 2 homeologous *TLC1* genes for the RNA component of telomerase that are present in the genome. Chromosome sequences that do not extend into telomeres instead terminate at gene families that are amplified in subtelomeric regions or contain genes that are at chromosome ends in the inferred Ancestral (pre-WGD) gene order for yeasts [41] indicating that they are almost full length, except for 3 chromosome ends that appear to have undergone break-induced replication (BIR) and homogenization with other chromosome ends.

We identified 1 scaffold as the mitochondrial genome, which maps as a 30-kb circle containing orthologs of all *S*. *cerevisiae* mitochondrial genes. We also found a plasmid in the 2-micron family (5,427 bp), with 99% sequence identity to pSB2, which was first isolated [42] from the type strain of *Z*. *parabailii* (NBRC1047/ATCC56075).

### *Z*. *parabailii* ATCC60483 is an interspecies hybrid, with *Z*. *bailii* as 1 parent

**Fig 1:**
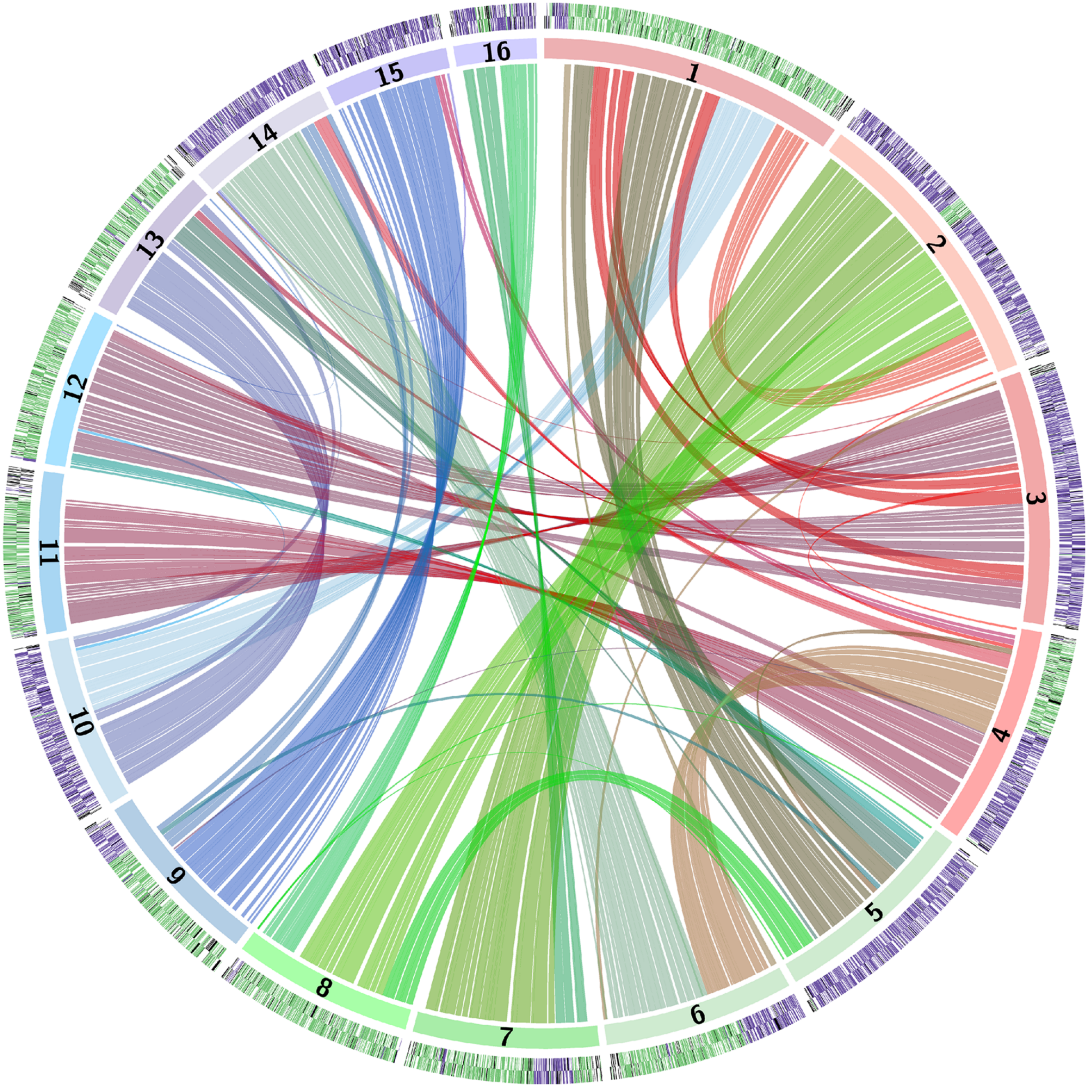
Circos plot of relationships among the *Z*. *parabailii* ATCC60483 chromosomes. In the outer arcs, purple and green coloring indicates A- and B-genes on the Watson and Crick strands of each chromosome. Arcs in the center of the diagram link homeologous (A:B) gene pairs.

Visualization of the genome using a Circos plot [43] shows that most of the genome is duplicated, indicating a polyploid origin (Fig 1). However, although most genes have a homeolog, the chromosomes do not form simple collinear pairs. Instead, sections of each chromosome are collinear with sections of other chromosomes.

**Fig 2:**
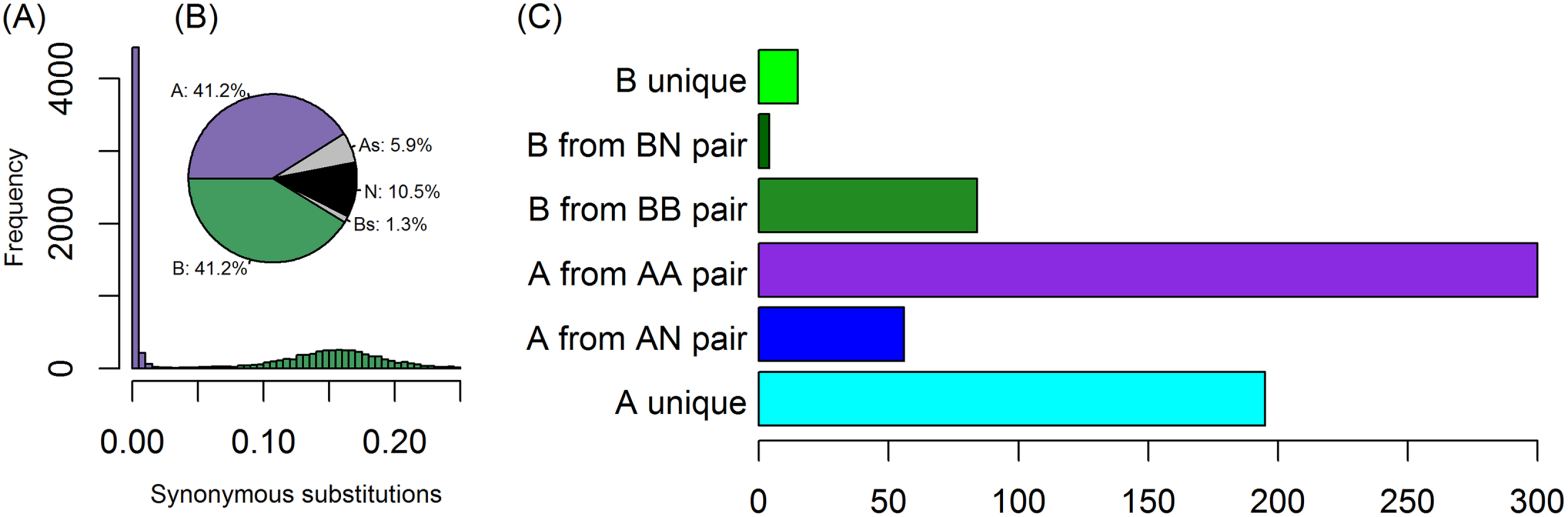
(A) Histogram of the distribution of synonymous site divergence (*K*_S_) values for 10,087 *Z*. *parabailii* ATCC60483 genes compared to their closest *Z*. *bailii* CLIB213^T^ homologs. (B) Pie chart showing the proportions of genes classified into each category. The 2 largest categories refer to A-genes and B-genes that are in A:B pairs. “N” means genes for which no *Z*. *bailii* homolog was found or *K*_S_ to *Z*. *bailii* exceeded 0.25. “As” and “Bs” indicate other A-genes and B-genes, as analyzed in panel C. (C) Breakdown of the numbers of genes assigned to the A- or B-subgenomes that are not in A:B pairs. See S1 Data for category counts and *K*_S_ values for each gene.

Comparison to *Z*. *bailii* CLIB213^T^ shows that for each region of the *Z*. *bailii* genome, there are 2 corresponding regions of the *Z*. *parabailii* genome: 1 almost identical in sequence and 1 with approximately 93% sequence identity, which demonstrates a hybrid (allopolyploid) origin of *Z*. *parabailii* and suggests that *Z*. *bailii* was one of its parents. To analyze this relationship in detail, we estimated the parental origin of every *Z*. *parabailii* ATCC60483 gene based on the number of synonymous substitutions per synonymous site (*K*_S_) when compared to its closest *Z*. *bailii* homolog (Fig 2A). This analysis revealed a bimodal distribution of *K*_S_ values in which 47.1% of the ATCC60483 genes are almost identical to CLIB213^T^ genes (*K*_S_ ≤ 0.05) and a further 42.5% are more divergent (0.05 < *K*_S_ ≤ 0.25).

From this relationship, we infer that *Z*. *parabailii* ATCC60483 is an interspecies hybrid formed by a fusion of 2 parental cells, which we refer to as Parent A (purple) and Parent B (green). Parent A was a cell with a genome essentially identical to *Z*. *bailii* CLIB213^T^. Parent B was a cell of an unidentified *Zygosaccharomyces* species with approximately 93% overall genome sequence identity to *Z*. *bailii*, corresponding to a synonymous site divergence peak of *K*_S_ = 0.16 (Fig 2A). We refer to the 2 sets of DNA in *Z*. *parabailii* that were derived from Parents A and B as the A-subgenome and the B-subgenome, respectively. We refer to the A- and B-copies of a gene as homeologs, and we use a suffix (“_A” or “_B”) in gene names to indicate which subgenome they come from.

The genome contains ribosomal DNA (rDNA) loci inherited from each of its parents. Our assembly includes 2 complete rDNA units with 26S, 5.8S, 18S, and 5S genes. Phylogenetic analysis of their internal transcribed spacer (ITS) sequences shows that the rDNA on chromosome 11 is derived from *Z*. *bailii* (Parent A), whereas the rDNA on chromosome 4 is derived from Parent B and contains an ITS variant seen only in other *Z*. *parabailii* strains (S1 Fig). A third rDNA locus in our assembly (at 1 telomere of chromosome 15) is incomplete and does not extend into the ITS region. The rDNA unit on chromosome 4 is also telomeric, whereas the unit on chromosome 11 is located at an internal site 165 kb from the right end. None of the genes in the interval between this rDNA and the right telomere of chromosome 11 have orthologs in *Z*. *bailii* CLIB213^T^.

*Z*. *parabailii* has 16 chromosomes. We identified its 16 centromeres bioinformatically, which correspond to 2 copies (A and B) of each of the 8 centromeres in the Ancestral pre-WGD yeast genome (Table 2) [41, 44]. In contrast, *Z*. *rouxii* has only 7 chromosomes because of a telomere-to-telomere fusion between 2 chromosomes followed by loss of a centromere [44]. The missing centromere in *Z*. *rouxii* is Ancestral centromere *Anc_CEN2*, which maps to *Z*. *parabailii* centromeres *CEN4* and *CEN11*, located between the genes *MET14* and *VPS1*. The *Z*. *rouxii* centromere must have been lost after it diverged from the *Z*. *bailii*/*Z*. *parabailii* lineage. Alignment of the *Z*. *rouxii MET14-VPS1* intergenic region with the *Z*. *parabailii CEN4* and *CEN11* regions shows that the CDE III motif of the point centromere has been deleted in *Z*. *rouxii* (S2 Fig).

**Table 2:** *Z*. *parabailii* ATCC60483 chromosomes and centromeres.

Chromosome	bp	Protein-coding genes	tRNA genes	Ancestral centromere^a^	*Z*. *rouxii* centromere
1	2,110,500	1,010	62	Anc_CEN5 (B)	Zr_CEN2
2	2,005,801	1,009	55	Anc_CEN6 (A)	Zr_CEN3
3	1,751,495	868	31	Anc_CEN4 (A)	Zr_CEN7
4	1,516,135	718	29	Anc_CEN2 (A)	absent^b^
5	1,443,312	709	44	Anc_CEN5 (A)	Zr_CEN2
6	1,315,104	614	22	Anc_CEN7 (B)	Zr_CEN4
7	1,283,838	638	29	Anc_CEN6 (B)	Zr_CEN3
8	1,249,162	634	28	Anc_CEN8 (B)	Zr_CEN6
9	1,240,939	579	43	Anc_CEN1 (B)	Zr_CEN5
10	1,189,704	576	28	Anc_CEN3 (A)	Zr_CEN1
11	1,115,933	522	16	Anc_CEN2 (B)	absent^b^
12	1,091,360	520	24	Anc_CEN4 (B)	Zr_CEN7
13	1,077,716	517	25	Anc_CEN3 (B)	Zr_CEN1
14	1,007,293	494	16	Anc_CEN7 (A)	Zr_CEN4
15	858,772	406	37	Anc_CEN1 (A)	Zr_CEN5
16	571,967	273	10	Anc_CEN8 (A)	Zr_CEN6
mtDNA	29,945	13	20		
Total (nuclear)	20,829,031	10087	499		

mtDNA, mitochondrial DNA.

^a^ Synteny correspondence between *Z*. *parabailii* centromeres and yeast Ancestral (pre–whole genome duplication [WGD]) centromere locations [44]. A and B indicate the subgenome assignments of the *Z*. *parabailii* centromeres.

^b^ *Z*. *rouxii* lost Anc_CEN2 in an evolutionary fusion of 2 chromosomes [44].

*Z*. *parabailii* inherited the mitochondrial genome of its *Z*. *bailii* parent. A complete mitochondrial genome sequence for *Z*. *bailii* is not available, but we identified 55 small mitochondrial DNA (mtDNA) contigs in the CLIB213^T^ assembly, which together account for most of the genome, and calculated an average of 96% sequence identity between these and ATCC60483 mtDNA. CLIB213^T^ lacks 2 of the 5 mitochondrial introns that are present in ATCC60483: the omega intron of the large subunit mitochondrial rDNA and intron 2 of *COX1*. Intraspecies polymorphism for intron presence/absence and comparable levels of intraspecies mtDNA sequence diversity have been reported in other yeast species [45, 46].

### Prehybridization chromosomal rearrangements in *Z*. *parabailii*’s parents relative to *Z*. *bailii*

**Fig 3:**
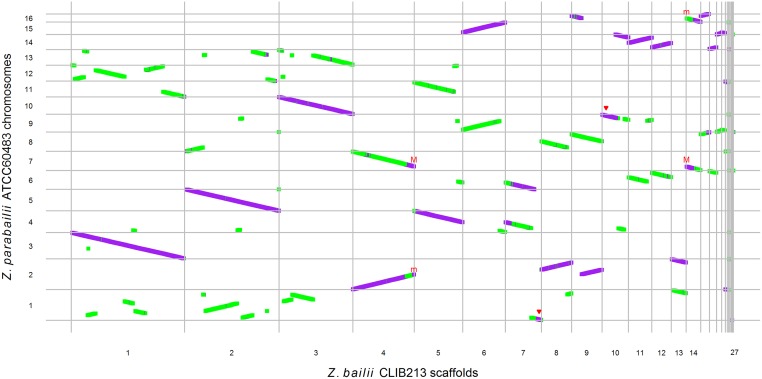
Dot-matrix plot between *Z*. *bailii* CLIB213^T^ scaffolds [38] and *Z*. *parabailii* ATCC60483 chromosomes. Each dot is a protein-coding gene (purple: A-genes; green, B-genes). Red triangles indicate chromosome ends that appear unpaired due to break-induced replication (BIR). “M” and “m” indicate the active and broken *MAT* loci of *Z*. *parabailii*, respectively.

When genes in the Circos plot are colored according to their parent of origin, it is striking that many *Z*. *parabailii* chromosomes are either almost completely “A” (purple) or almost completely “B” (green) (outer ring in Fig 1), even though the chromosomes do not form collinear pairs. This pattern can be seen in more detail in a dot-matrix plot between *Z*. *bailii* and *Z*. *parabailii* (Fig 3). From this plot, it is evident that most of the A-subgenome is collinear with *Z*. *bailii* scaffolds, whereas the B-subgenome contains many rearrangements relative to *Z*. *bailii*. For example, *Z*. *parabailii* chromosome 1 is derived almost entirely from the B-subgenome but maps to about 12 different regions on the *Z*. *bailii* scaffolds. In contrast, *Z*. *parabailii* chromosome 3 is derived from the A-subgenome and is collinear with a single *Z*. *bailii* scaffold.

In total, from Fig 3 we estimate that there are approximately 34 breakpoints in synteny between the *Z*. *parabailii* B-subgenome and *Z*. *bailii* but no breakpoints between the A-subgenome and *Z*. *bailii*, when posthybridization rearrangement events (described below) are excluded. This difference in the levels of rearrangement in the A- and B-subgenomes relative to *Z*. *bailii* indicates that the 2 subgenomes were not collinear at the time the hybrid was formed. Therefore, most of the rearrangements between the 2 subgenomes are rearrangements that existed between the 2 parental species prior to hybridization. The 2 parents both had 8 chromosomes, but their karyotypes were quite different. Because each event of reciprocal translocation or inversion creates 2 synteny breakpoints [47], we estimate that about 17 events of chromosomal translocation or inversion occurred between the 2 parents in the time interval between when they last shared a common ancestor and when they hybridized. The situation in *Z*. *parabailii* (hybridization between parents differing by 17 rearrangements and 7% sequence divergence) contrasts with that in the hybrid *Millerozyma sorbitophila* (only 1 detectable rearrangement between the parents, despite 15% sequence divergence [13]).

### Posthybridization recombination, loss of heterozygosity (LOH), and BIR

Although the *Z*. *parabailii* genome largely contains unrearranged parental chromosomes, there have been 2 major types of rearrangement after hybridization. First, posthybridization recombination between the subgenomes at homeologous sites has formed some chromosomes that are partly “A” and partly “B.” Second, a process of homogenization has occurred at some places in which 1 subgenome overwrote the other, resulting in gene pairs that are A:A or B:B. This process is commonly called loss of heterozygosity (LOH) or gene conversion. Based on their *K*_S_ distances from *Z*. *bailii*, the genome contains 4,153 simple A:B homeologous gene pairs, 300 A:A pairs, and 84 B:B pairs.

**Fig 4:**
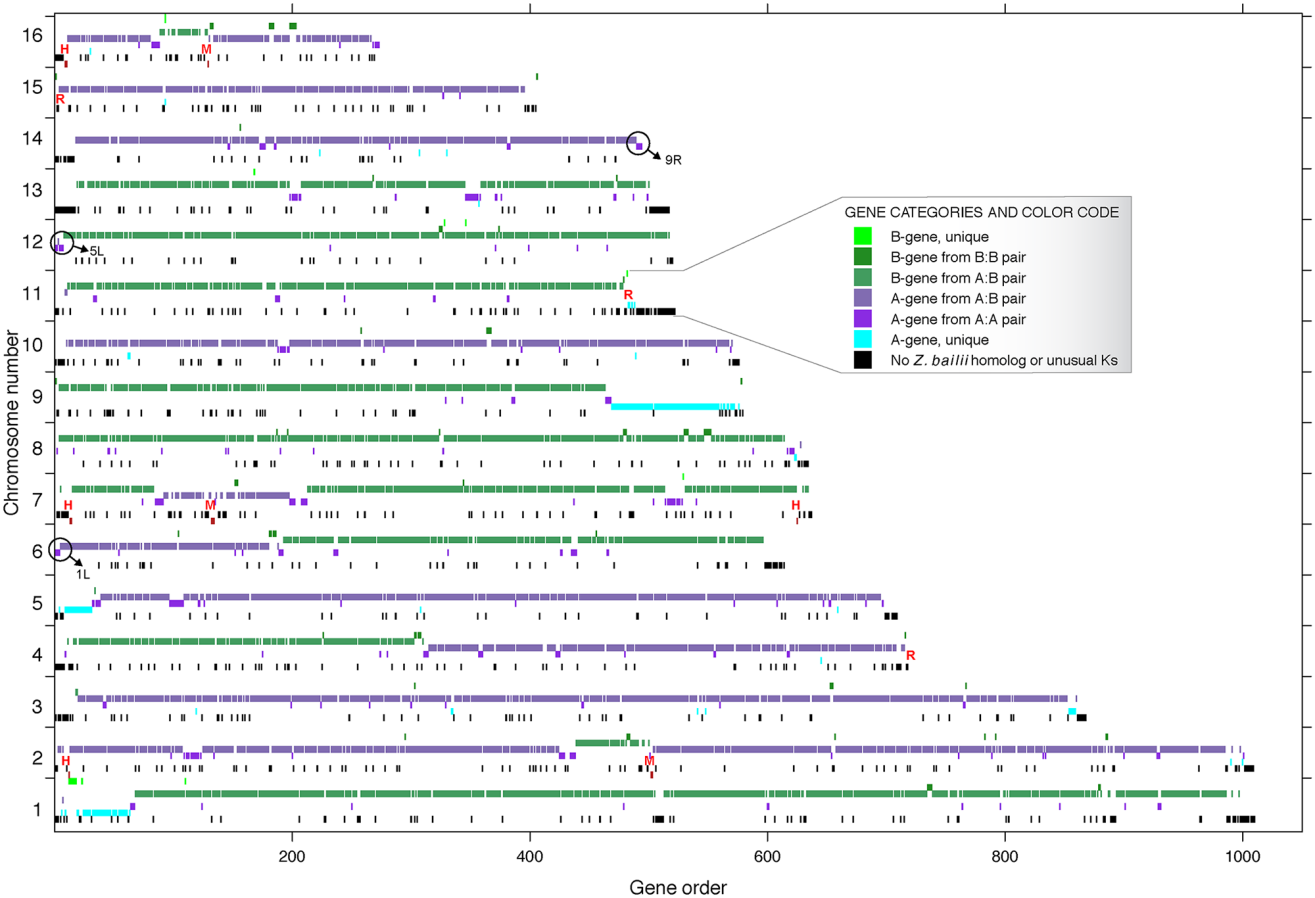
Subgenome and duplication status of each *Z*. *parabailii* gene. Each gene was classified into 1 of 7 categories and color-coded as shown in the legend. For each chromosome, 7 rows were then drawn, showing the locations of genes in each category (the 7 rows appear in the same order from top to bottom as in the legend). “R” shows the locations of ribosomal DNA (rDNA clusters). “M” and “H” indicate the locations of *MAT* and *HML*/*HMR* loci. Circles with arrows mark the 3 chromosome ends where our sequence is incomplete due to break-induced replication (BIR); in each case, the missing sequence is apparently identical to the end of another chromosome, as shown. For example, we infer that at the right end of chromosome 14, our assembly artefactually lacks a second copy of the genes that are labeled as “A unique” on the right end of chromosome 9. The high sequence identity of the chromosome 9 and 14 copies of this region caused them to coassemble, and the coassembled contig was arbitrarily assigned to chromosome 9.

To examine the genomic locations of LOH and rearrangement events in more detail, we further classified genes using a scheme that takes account of their pairing status as well as their divergence from *Z*. *bailii*. Genes were defined as “A” or “B” as before or “N” if a *K*_S_ distance from *Z*. *bailii* could not be calculated (Fig 2B and 2C). We then assigned each gene to 1 of 7 categories such as “B-gene in an A:B pair” or “A-gene, unpaired” and plotted the locations of genes in each category. The resulting map of the genome (Fig 4) allows LOH and recombination events to be visualized. N-genes (black in Fig 4) are seen to be mostly located near telomeres. Several points of recombination between the A- and B-subgenomes are apparent, such as in the middle of chromosome 4. LOH tends to occur in stretches that span multiple genes. For example, on chromosome 13, LOH has formed 8 runs of consecutive A-genes in a chromosome that is otherwise “B”; these A-genes are members of A:A pairs. They were probably formed by homogenization (gene conversion without crossover), although they could also be the result of double crossovers followed by meiotic segregation of chromosomes. Patches of LOH are frequently seen adjacent to sites of recombination between the 2 subgenomes (Fig 4). Three large regions of apparently unpaired A-genes near the ends of chromosomes (1L, 5L, and 9R; light blue in Fig 4) are probably artefacts caused by BIR, which is a process that can make the ends of 2 chromosomes completely identical from an initiation point out to the telomere [48]. These regions have 2x sequence coverage in our Illumina data, and we can identify the probable locations of an identical second copy of each of them at other chromosome ends (Fig 4).

### Rearrangement catalyzed by HO endonuclease and degeneration of the “B” *MAT* locus

**Fig 5:**
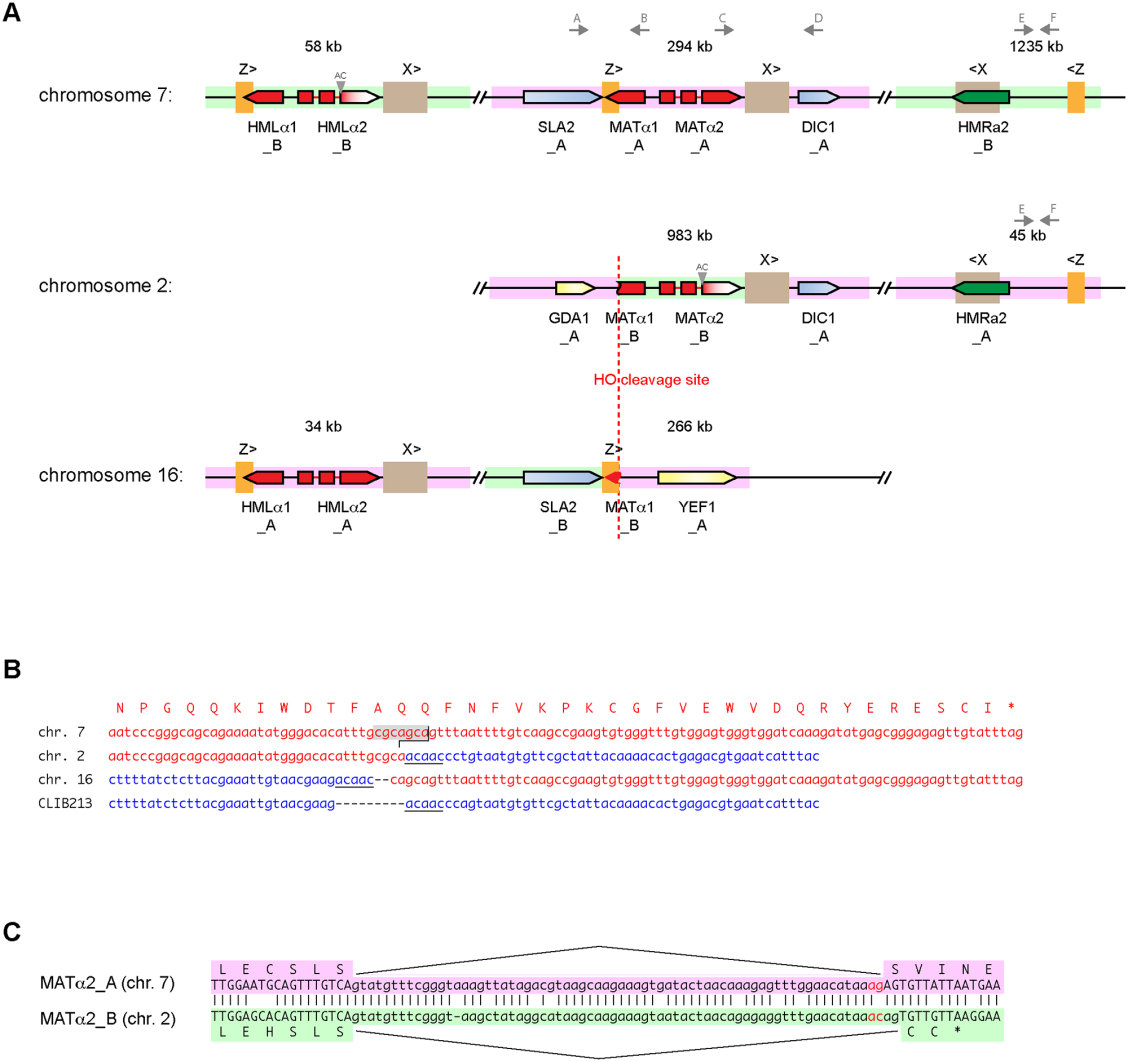
(A) Organization of *MAT*, *HML*, and *HMR* loci in *Z*. *parabailii* ATCC60483. The genome contains 6 *MAT*-related regions, with 1 *MAT*, 1 *HML*, and 1 *HMR* locus derived from each of the A and B parents. Pink and green backgrounds indicate sequences from the A- and B-subgenomes, respectively. The *MAT* locus in the A-subgenome (position 294 kb on chromosome 7) is intact and expressed. The *MAT* locus of the B-subgenome has been broken into 2 parts by cleavage by HO endonuclease. All 6 copies of the X repeat region (654 bp) are identical in sequence, as are all 6 copies of the Z repeat region (266 bp). Gray triangles indicate the disruption of the splicing of intron 2 in *MAT*α2 and *HML*α2 of the B-subgenome. The binding sites for primers A–F used for PCR amplification are indicated by gray arrows. (B) Sequences at the *MAT* locus breakpoint. Red, *MAT*α1-derived sequences. The HO cleavage site (CGCAGCA, giving a 4-nucleotide 3′ overhang) is highlighted in gray. Blue, the *GDA1-YEF1* intergenic region from the equivalent region of *Z*. *bailii* CLIB213^T^ and homologous sequences from the A-subgenome on *Z*. *parabailii* chromosomes (chrs.) 2 and 16. A 5-bp sequence (ACAAC) that became duplicated during the rearrangement is underlined. (C) Sequences of *MAT*α2 intron 2 (lowercase) from the A- and B-subgenomes. An AG-to-AC mutation (red) at the 3′ end of the intron moved the splice site by 2 bp in the B-subgenome, causing a frameshift and premature translation termination. The splice sites in both genes were identified from RNA sequencing (RNA-Seq) data.

The *Z*. *parabailii* genome contains 2 *MAT* loci (one of which is broken) and 4 *HML*/*HMR* silent loci (Fig 5). In *S*. *cerevisiae*, mating-type switching is a DNA rearrangement process that occurs in haploid cells to change the genotype of the *MAT* locus [49]. During switching, the active *MAT* locus is first cleaved by an endonuclease called HO, and its **a**- or α-specific DNA is removed by an exonuclease. The resulting double-strand DNA break at *MAT* is then repaired by copying the sequence of either the *HML*α or *HMR***a** locus. This process converts a *MAT***a** genotype to *MAT*α, or vice versa. Repeated sequences, called Z and X, located beside *MAT* and the *HM* loci act as guides for the DNA strand exchanges that occur during this repair process. The *HM* loci are “silent” storage sites for the **a** and α sequence information because genes at these loci are not transcribed due to chromatin modification; only *MAT* is transcribed [49].

We infer that the parents of *Z*. *parabailii* each contained a *MAT* locus and 2 silent loci (*HML*α and *HMR***a**), similar to *S*. *cerevisiae* and *Z*. *rouxii* haploids [50]. Fig 5A shows that *Z*. *parabailii* has a *MAT* locus on chromosome 7, flanked by Z and X repeats and full-length copies of the genes *SLA2* and *DIC1*, similar to the *MAT* loci of many other species [50, 51]. This *MAT* locus is derived from Parent A. Chromosome 7 also contains *HML*α and *HMR***a** loci (derived from Parent B) near its telomeres. However, the B-subgenome’s *MAT* locus is broken into 2 pieces. Most of it is on chromosome 2, but its left part (the 3′ end of *MAT*α1, the Z repeat, and the neighboring gene *SLA2*) is on chromosome 16 (Fig 5A). Chromosomes 2 and 16 also each contain an *HML*α or *HMR***a** locus from the A-subgenome.

Examination of the breakpoint in the B-subgenome’s *MAT* locus shows that the break was catalyzed by HO endonuclease, because it occurs precisely at the cleavage site for this enzyme (Fig 5B). In *S*. *cerevisiae*, HO has a long (approximately 18 bp) recognition sequence that is unique in the genome, and it cleaves DNA at a site within this sequence, leaving a 4-nucleotide 3′ overhang [52]. Although the recognition and cleavage sites of HO endonucleases in other species have not been investigated biochemically, they can be deduced because the core of the HO cleavage site (cgcagca) invariably forms the first nucleotides of the Z region in each species [51]. Moreover, the HO cleavage site corresponds to an amino acid sequence motif (faqq) in the *MAT*α1 protein that is strongly conserved among species.

The 2 parts of the broken *MAT* locus are located beside the genes *GDA1* and *YEF1* (Fig 5A), which are neighbors in *Z*. *bailii* CLIB213^T^ and in the Ancestral yeast genome [38, 41]. Therefore, after HO endonuclease cleaved the “B” *MAT* locus, the broken ends of the chromosome apparently interacted with the *GDA1-YEF1* intergenic region of the A-subgenome, causing a reciprocal translocation. This site is the only synteny breakpoint between the A-subgenome of *Z*. *parabailii* and the genome of *Z*. *bailii* (scaffold 9; Fig 3). Comparison of the DNA sequences at the site (Fig 5B) shows no microhomology between the 2 interacting sequences and that DNA repair led to duplications of a 5-bp sequence (acaac) from the *GDA1-YEF1* intergenic region and a 2-bp sequence (ca) from *MAT*α1, suggestive of nonhomologous end joining (NHEJ) as the repair mechanism. We hypothesize that this genomic rearrangement occurred during a failed attempt to switch mating types, which resulted in a reciprocal translocation instead of normal repair of *MAT* by *HML* or *HMR*.

While the B-subgenome’s *MAT*α1 gene is clearly broken, its *MAT*α2 gene also appears to be nonfunctional. *MAT*α2 has 2 introns, and our RNA-Seq data show how both homeologs of this gene (*ZPAR0G01480_A* and *ZPAR0B05090_B*) are spliced. A point mutation at the 3′ end of intron 2 of the B-gene changed its AG splice acceptor site to AC, with the result that splicing now uses another AG site 2 nucleotides downstream (Fig 5C). This change results in a frameshift, truncating the B-copy of the α2 protein to 57 amino acid residues instead of 211 and presumably inactivating it.

Surprisingly, the *Z*. *parabailii* genome does not contain any *MAT***a**1 (or *HMR***a**1) gene. This gene codes for the **a**1 protein, which is 1 subunit of the heterodimeric **a**1-α2 transcriptional repressor that is formed in diploid (**a**/α) cells and which acts as a sensor of diploidy by repressing transcription of haploid functions such as mating while permitting diploid functions such as meiosis [53]. The **a**1 gene is present in *Z*. *rouxii* and *Z*. *sapae* [27, 37, 50], but it is also absent from *Z*. *bailii* CLIB213^T^ and must have been absent from Parent B. The *Z*. *bailii* CLIB213^T^ *MAT* organization is not fully resolved [38], but it contains a *MAT* locus with α1 and α2 genes on scaffold 14 and an *HMR* locus with only an **a**2 gene on scaffold 19. Evolutionary losses of *MAT***a**1 have previously been seen in some *Candida* species [54, 55], but not in any species of family Saccharomycetaceae. In contrast, the gene for the other subunit of the heterodimer, *MAT*α2, is present in all *Zygosaccharomyces* species and is probably maintained because it has a second role in repressing **a**-specific genes in this genus [56]. Solieri and colleagues have reported evidence that **a**1-α2 is nonfunctional in a *Z*. *rouxii/pseudorouxii* hybrid in which its 2 subunits are derived from different species [14].

### *Z*. *parabailii* strains ATCC60483 and ISA1307 are descendants of the same interspecies hybridization event

The 2 subgenomes apparent in the Illumina scaffolds of the *Zygosaccharomyces* hybrid strain ISA1307, previously sequenced by Mira et al. [39], are both 99%–100% identical in sequence to the A- or B-subgenomes of ATCC60483. Therefore, ISA1307 is also a strain of *Z*. *parabailii*. Importantly, the ISA1307 genome sequence contains the same HO-catalyzed reciprocal translocation between *MAT*α1 of the B-subgenome and the *GDA1-YEF1* intergenic region of the A-subgenome (Fig 5A). Because this rearrangement is so unusual and because it did not involve recombination between repeated sequences, it is highly unlikely to have occurred twice in parallel. The rearrangement is much more likely to have occurred only once, in a common ancestor of the 2 *Z*. *parabailii* strains after the hybrid was formed. It cannot pre-date the hybridization because it formed junctions between the A- and B-subgenomes, which originated from different parents.

ATCC60483 and ISA1307 are independent isolates of *Z*. *parabailii*, both from industrial sources. ATCC60483 was isolated from citrus concentrate used for soft drink manufacturing in the Netherlands [57, 58], and ISA1307 was a contaminant in a sparkling wine factory in Portugal [39, 59–61]. We found several examples in which the 2 strains differ in their patterns of LOH, which confirms that they have had some extent of independent evolution. All 3 large regions of BIR (on chromosomes 1, 5, and 9; Fig 4) are unique to ATCC60483. ISA1307 contains A:B homeolog pairs throughout these regions, whereas ATCC60483 has only A-genes, which we infer to be in A:A pairs. Other examples of differential LOH include a 4-kb region around homologs of the *S*. *cerevisiae* gene *YLR049C*, which exists as B:B pairs in ATCC60483 but A:B pairs in ISA1307, and the gene *KAR4*, which is an A:B pair in ATCC60483 but only a B-gene (single contig) in ISA1307. Notably, the section of the *RPB1* gene (also called *RPO21*) that Suh et al. [28] used for taxonomic identification of *Z*. *parabailii* and *Z*. *pseudobailii* exists as an A:B pair in ATCC60483, but only as an A-gene in the ISA1307 genome. The absence of the B-copy of *RPB1* made Mira et al. [39] hesitant to conclude that ISA1307 is *Z*. *parabailii*.

### *Z*. *parabailii* ATCC60483 is fertile and haploid

**Fig 6:**
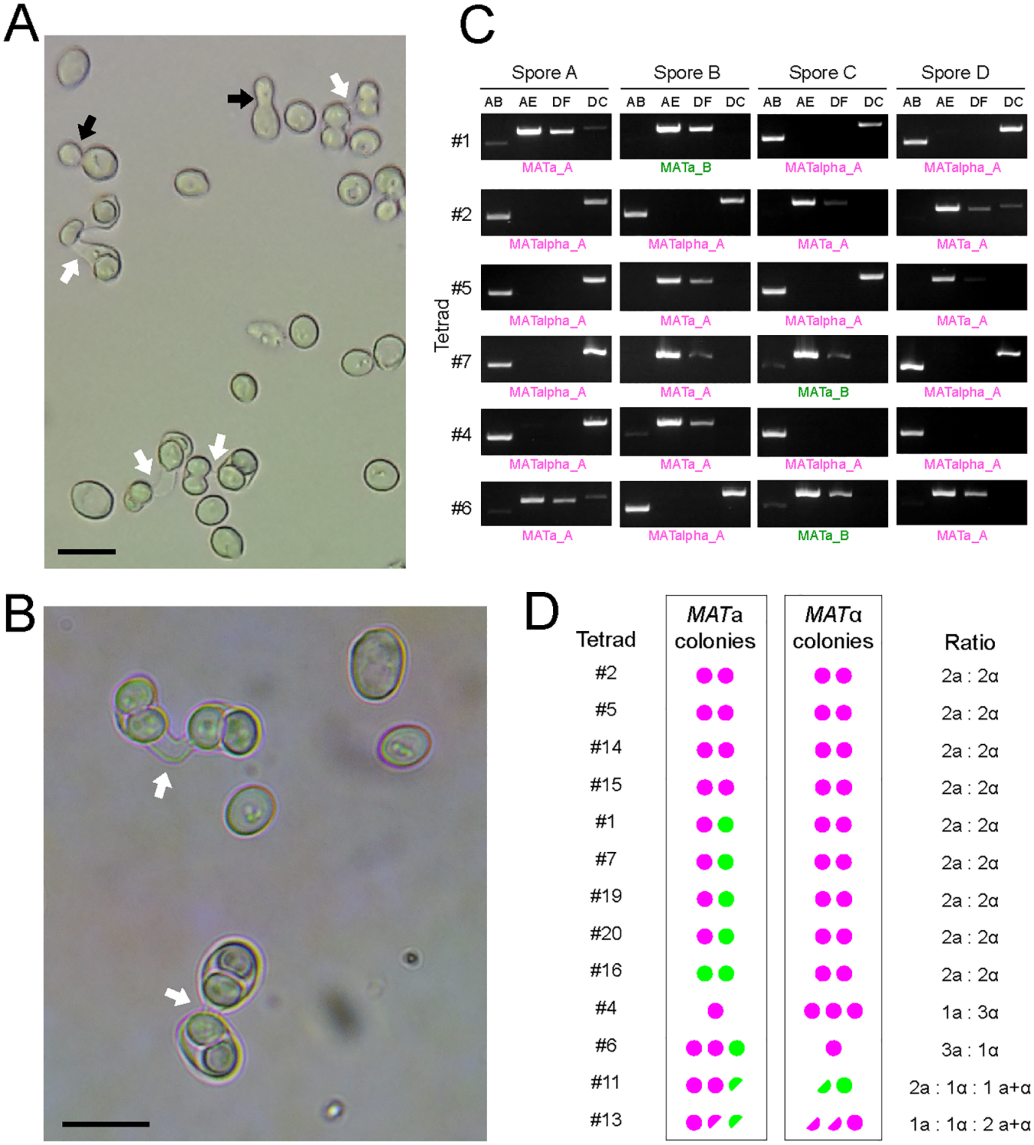
(A,B) Ascospore formation in *Z*. *parabailii* ATCC60483. White arrows show conjugation tubes in dumbbell-shaped asci. Black arrows show budding vegetative cells. Scale bars, 10 μm. Cultures were grown on 5% malt extract agar for 6–10 days at 25°C. (C) Examples of PCR determination of *MAT* locus genotypes in tetrads. Pairs of PCR primers as shown in Fig 5A were used to amplify the *MAT* locus in colonies grown from spores after dissection of conjugated asci. PCR primer pairs AB and AE amplify the left side of the *MAT* locus, including the Z region (AB, 1,485-bp product from *MAT*α; AE, 2,103-bp product from *MAT***a**). Primer pairs DF and DC amplify the right side of the *MAT* locus, including the X region (DF, 1,882-bp product from *MAT***a;** DC, 2,027-bp product from *MAT*α). PCR products were sequenced to determine whether they originated from the A- or B-subgenome. (D) Summary of *MAT* genotypes in colonies grown from spores from 13 dissected tetrads. Magenta circles denote colonies with A-subgenome alleles (*MAT***a**_A or *MAT*α_A), and green circles denote colonies with B-subgenome alleles (*MAT***a**_B or *MAT*α_B). Half circles represent colonies that gave both *MAT***a** and *MAT*α PCR products.

Both ATCC60483 and the type strain of *Z*. *parabailii* ATCC56075^T^ have previously been reported to be capable of forming ascospores [28, 57, 58]. We confirmed that our stock of ATCC60483 is able to sporulate (Fig 6A and 6B). On malt extract agar plates, we observed that sporulation occurs directly in zygotes formed by conjugation between 2 cells, resulting in asci in which the 2 former parental cell bodies typically contain 2 ascospores each. Such dumbbell-shaped (conjugated) asci, indicative of sporulation immediately after mating, are characteristic of the genus *Zygosaccharomyces* [25] and have previously been described in other *Z*. *bailii* (sensu lato) strains [25, 62–66]. The presence of conjugating cells in a culture grown from a single strain indicates that ATCC60483 is functionally haploid (capable of mating) and that it is homothallic (capable of mating-type switching). Since the zygote proceeds immediately into sporulation without further vegetative cell divisions, the diploid state of *Z*. *parabailii* appears to be unstable. Although Suh et al. [28] reported that asci of the type strain of *Z*. *parabailii* contain 2 spores, we consistently observed that asci occur in pairs of mated cells connected by a conjugation tube (Fig 6A and 6B), indicating that 4 spores are formed per meiosis.

We dissected tetrad asci from ATCC60483, grew colonies from the spores, and then used colony PCR to determine their genotype at the intact *MAT* locus on chromosome 7. Among 13 tetrads analyzed, 9 showed a ratio of 2 *MAT***a** colonies to 2 *MAT*α colonies (Fig 6C and 6D). Two tetrads showed 1:3 or 3:1 ratios, and the other two yielded both *MAT***a** and *MAT*α PCR products from some single-spore colonies. The genotype of the ATCC60483 starting strain is *MAT*α from the A-subgenome (designated *MAT*α_A), so the presence of *MAT***a** genotypes in colonies derived from spores made by this strain confirms that mating-type switching occurred at some point. We sequenced the PCR products and found that the A- and B-subgenome *HMR***a** loci were both used as donors for mating-type switching: among the pure *MAT***a** colonies, 18 were *MAT***a**_A, and 7 were *MAT***a**_B (Fig 6D). Quite surprisingly, 4 tetrads with 2**a**:2α segregation had 1 *MAT***a**_A and 1 *MAT***a**_B spore colony, which is inconsistent with simple meiotic segregation from an **a**/α diploid. Because all the spores contain a functional *HO* gene, the genotypes of these 4 tetrads (#1, #7, #19, and #20) probably result from additional switches during the early growth of some colonies. Similarly, switching during early colony growth may explain the presence of *MAT*α_B genotypes in tetrad #11 and the colonies with mixed **a**+α genotypes (in tetrads #11 and #13), as well as the presence of faint PCR products corresponding to the alternative *MAT* genotype in some other colonies (Fig 6C). In *S*. *cerevisiae*, homothallic diploid (*HO/HO MAT***a**/*MAT*α) strains show 2:2 segregation of *MAT* alleles in tetrads, but after spore germination the haploid cells can then switch mating types as often as once per cell division [67], leading to mating and colonies that contain mostly diploid cells [68]; by contrast, most (but not all) of the *Z*. *parabailii* spore-derived colonies contained a single mating type (Fig 6C and 6D).

We found that almost all the genes involved in mating and meiosis that Mira et al. [39] reported to be missing from the *Z*. *parabailii* ISA1307 genome are in fact present in both ATCC60483 and ISA1307 (S1 Table). For example, we annotated A- and B-homeologs of *IME1*, *UME6*, *DON1*, *SPO21*, *SPO74*, *REC104*, and *DIG1*/*DIG2* as well as *MAT***a**2, *MAT*α1, and *MAT*α2. We also identified genes for the α-factor and **a**-factor pheromones (*MF*α and *MF*a). The *MF*α genes code for an unusually high number of copies (10–14) of a 13-residue peptide whose consensus sequence, ahlvrlspgaamf, is quite different from that of other yeasts, including *Z*. *rouxii* (7/13 matches) and *S*. *cerevisiae* (4/13 matches) [2]. *Z*. *parabailii* and *Z*. *bailii* do lack most of the ZMM group of genes, involved in crossover interference during recombination [69], even though these are present in *Z*. *rouxii* (S1 Table). Interestingly, identical sets of ZMM genes have been lost in *Z*. *bailii*/*Z*. *parabailii* relative to *Z*. *rouxii*, as were lost in most *Lachancea* species relative to *Lachancea kluyveri* [70]: *ZIP2*, *CST9 (ZIP3)*, *SPO22 (ZIP4)*, *MSH4*, *MSH5*, and *SPO16* are absent, as well as *MLH2*, which is not known to be a ZMM gene, whereas *ZIP1* is retained. A similar loss of ZMM genes has occurred in *Eremothecium gossypii* relative to *E*. *cymbalariae* [71].

### Posthybridization gene inactivations

A small number of *Z*. *parabailii* ATCC60483 genes have “disabling” mutations—frameshifts or premature stop codons that prevent translation of a normal protein product. The majority of these mutations are present in only 1 subgenome of ATCC60483 and are unique to this strain. For example, there is a 1-bp insert in the A-homeolog of the DNA repair gene *MLH1* that is not present in the B-homeolog or in ISA1307 or CLIB213^T^. In a systematic search, we found a total of 10 A-genes and 9 B-genes that were inactivated only in strain ATCC60483 (S2 Table). In each case, the other homeolog was intact, and the mutations, discovered in the PacBio assembly, were confirmed by our Illumina contigs of the ATCC60483 genome.

We found a further 8 disabling mutations that are shared between ATCC60483 and ISA1307. One of these is the AC-to-AG splice site mutation in the B-homeolog of *MAT*α2 described above (Fig 5C). Another is the *HO* endonuclease gene, whose A-homeolog contains an identical 1-bp deletion in both ATCC60483 and ISA1307, whereas the B-homeolog of *HO* is intact in both strains (S2 Table). It is perhaps surprising that the *HO* gene that degenerated is the A-homeolog, whereas the broken *MAT* locus is the B-homeolog, but the 2 endonucleases are likely to have had identical site specificities because the HO cleavage site is well conserved among species. The existence of these 8 shared disabling mutations provides further support for the idea that the 2 strains of *Z*. *parabailii* are descended from the same hybrid ancestor, because these mutations may not be viable in the absence of the intact homeologous copies of these genes. Only one of them is present also in CLIB213^T^ (S2 Table).

### In-frame introns and other features of the genome

We annotated 447 introns in the *Z*. *parabailii* ATCC60483 genome, most of which are confirmed by our RNA-Seq data. There are 428 intron-containing genes, including 19 that have 2 introns. We did not find any examples of intron presence/absence differences between homeologs. Interestingly, we found several genes with an in-frame intron—that is, an intron that is a multiple of 3 bp long and contains no stop codons, so that both the spliced and unspliced forms of the mRNA can be translated into proteins. Genes with in-frame introns are likely to undergo alternative splicing, making 2 forms of the protein with different functions. One of these loci is *PTC7* (*ZPAR0J04940_A* and *ZPAR0A06900_B*). Both of the *Z*. *parabailii* homeologs contain a 69-bp in-frame intron within the open reading frame (ORF) of the gene. It has previously been shown that alternative splicing of a similar in-frame intron in *S*. *cerevisiae PTC7* leads to the translation of a mitochondrial protein isoform from the spliced mRNA and a nuclear envelope protein isoform from the unspliced mRNA and that the intronic region codes for a transmembrane domain of the protein [72]. Thus, the alternative splicing mechanism in *PTC7* is conserved between *Saccharomyces* and *Zygosaccharomyces*. We also found in-frame introns in the *Z*. *parabailii* orthologs of *S*. *cerevisiae NUP100*, *NCB2*, and *HEH2*, identically in their A- and B-homeologs. None of these genes is known to be alternatively spliced in *S*. *cerevisiae*. In each of these examples, there are typical splice donor, branch, and acceptor sequences within the long form of the ORF.

Programmed “+1” ribosomal frameshifting, a process whereby the ribosome skips forward by 1 nucleotide when translating an mRNA, is known to occur in 3 genes in *S*. *cerevisiae*: *OAZ1*, *ABP140*, and *EST3* [73], and we found that +1 frameshifting is also required to translate the *Z*. *parabailii* orthologs of these 3 genes, in both the A- and B-homeologs. We also found 2 new loci that apparently undergo +1 frameshifting. Translation of both homeologs of *BIR1* (*ZPAR0O02690_A* and *ZPAR0I02720_B*) requires a +1 frameshift at a sequence identical to the *EST3* frameshifting site: CTT-A-GTT, where the A is the skipped nucleotide. Translation of both homeologs of *YJR112W-A* (*ZPAR0O02960_A*, *ZPAR0I02990_B*) requires a +1 frameshift at a sequence identical to the *ABP140* frameshifting site: CTT-A-GGC.

In *S*. *cerevisiae*, the *CUP1* locus confers resistance to copper toxicity by a gene amplification mechanism. *CUP1* codes for a metallothionein, a tiny cysteine-rich copper-binding protein. The reference *S*. *cerevisiae* genome sequence contains 2 identical copies of *CUP1* duplicated in tandem, but under copper stress this locus can become amplified to contain up to 18 tandem copies of the gene [74, 75]. There are at least 5 different types of *CUP1* repeats in different *S*. *cerevisiae* strains, which must have originated independently from progenitors with a single *CUP1* gene [75, 76]. In *Z*. *parabailii*, we found a slightly different organization. At homeologous loci on chromosomes 2 and 7, ATCC60483 has multiple identical copies of a 1,454-bp repeating unit. Each unit contains 2 metallothionein genes, *MT-58* and *MT-47*, coding for proteins of 58 and 47 residues, respectively. There is only 56% amino acid sequence identity between MT-58 and MT-47 proteins. The chromosome 7 locus contains 5 copies of the repeating unit, and the chromosome 2 locus contains 2 copies, so ATCC60483 has 14 metallothionein genes in total. These loci are not syntenic with *S*. *cerevisiae CUP1*, but they are syntenic with metallothionein genes in *C*. *glabrata* and *Z*. *rouxii* [77, 78].

## Discussion

Our results show that *Z*. *parabailii* is a hybrid species that was formed by fusion between two 8-chromosome parental species, one of which was *Z*. *bailii*. The low sequence divergence of the ATCC60483 A-subgenome from the type strain of *Z*. *bailii* (the modal synonymous site divergence is less than 1%; Fig 2A) and the almost complete collinearity of these genomes (Fig 3) indicate that the A-parent of *Z*. *parabailii* should be regarded as *Z*. *bailii* itself, and not merely as a species closely related to *Z*. *bailii*.

**Fig 7:**
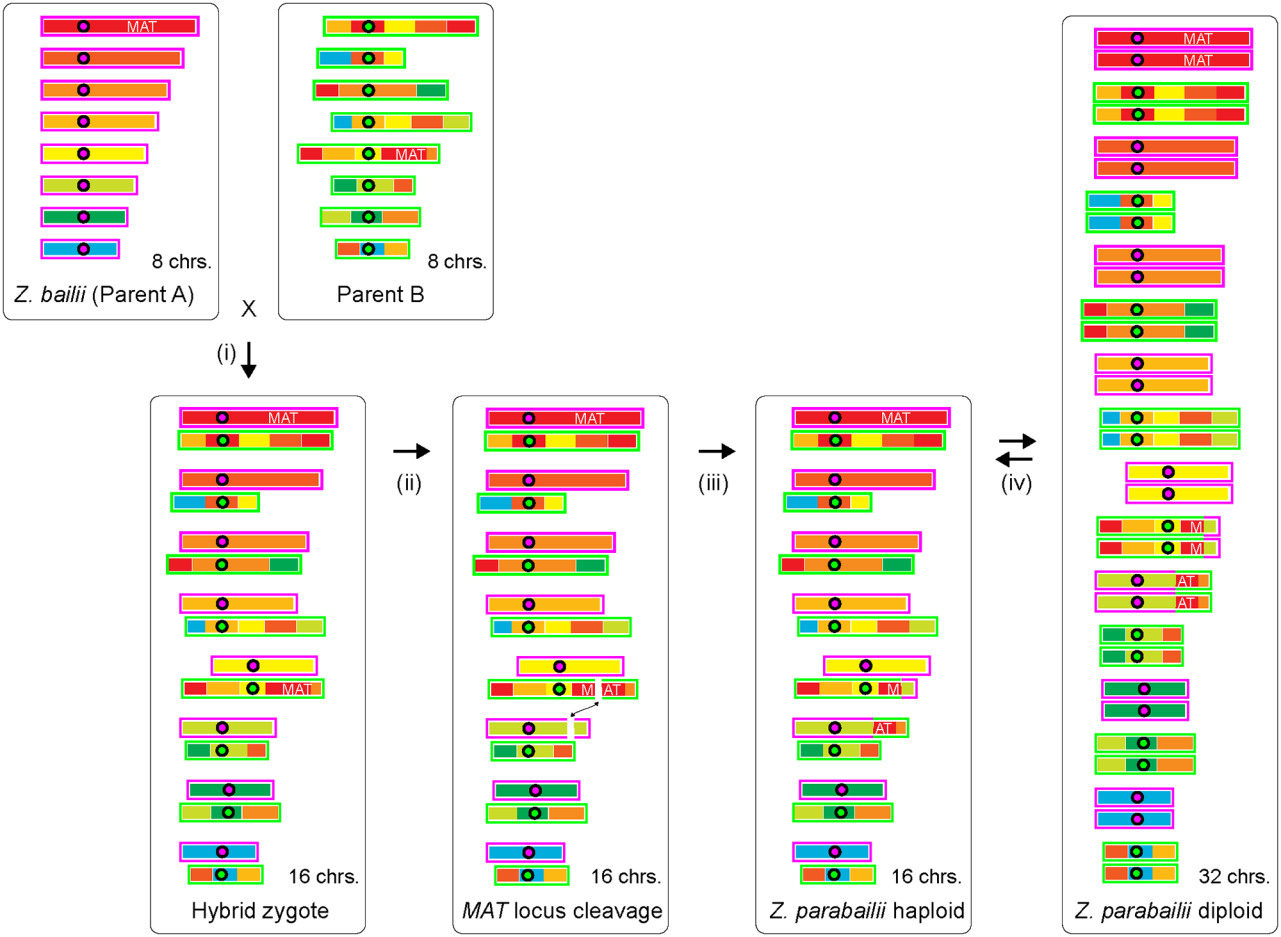
Cartoon of key steps in the origin of the *Z*. *parabailii* genome. Chromosome regions (thick bars) are colored according to their location in *Z*. *bailii* (magenta outlines). The corresponding homeologous regions are scrambled in Parent B (green outlines). Circles represent centromeres. (i) Interspecies mating occurred between Parent A (*Z*. *bailii*) and Parent B. The genomes differed by about 34 rearrangement breakpoints and 7% nucleotide sequence divergence. The resulting zygote was unable to form viable spores because of the noncollinearity of its chromosomes. (ii) Expression of HO endonuclease in the zygote, due to the absence of **a**1-α2, resulted in cleavage of the B-copy of the *MAT* locus and ectopic recombination with the *GDA1-YEF1* region of the A-subgenome, causing a reciprocal translocation. (iii) The resulting genome has only 1 functional *MAT* locus and behaves as a haploid. Recombinations and other exchanges between homeologous regions of the 2 subgenomes, such as those that exchanged the *HML*/*HMR* regions, occurred but are not shown here for simplicity. (iv) The current life cycle of *Z*. *parabailii* involves mating between 16-chromosome haploids to form 32-chromosome diploids, which immediately sporulate to regenerate 16-chromosome haploids. *Z*. *parabailii* is homothallic because it contains an intact *HO* gene, which allows interconversion between *MAT***a** and *MAT*α haploids and hence autodiploidization. chrs., chromosomes.

The unusual *MAT* locus structure of this hybrid raised questions about how it was formed and whether *Z*. *parabailii* currently has a full sexual cycle. At first glance, the *MAT*α/*MAT*α hybrid genotype of ATCC60483 might suggest that *Z*. *parabailii* could not have been formed by mating. However, this genotype could also be the result of mating-type switching. We propose that the following steps occurred (Fig 7). *Z*. *parabailii* was formed by mating between strains of parent A (*Z*. *bailii*) and parent B, of opposite mating types. These parental genomes already differed by about 34 chromosomal rearrangement breakpoints, so the hybrid was unable to produce viable spores by meiosis. The hybrid also had no *MAT***a**1 gene, so it could not form the **a**1-α2 heterodimer that stabilizes the diploid state in *S*. *cerevisiae* [68]. One of the roles of the **a**1-α2 dimer in *S*. *cerevisiae* is to repress transcription of *HO* endonuclease, which is only required in haploid cells. We suggest that in the newly formed *Z*. *parabailii* hybrid, transcription of *HO* was not repressed. Continued expression of this gene resulted in genotype switching at the *MAT* loci (perhaps several consecutive switches between **a** and α) and, eventually, breakage of the B-subgenome *MAT* locus due to an illegitimate recombination with the *GDA1-YEF1* intergenic region instead of *HML* or *HMR*. At some point after hybridization, the *HO* gene from the A-subgenome also degenerated by acquiring a frameshift mutation.

The breakage of the “B” *MAT* locus can be inferred to have been one of the first rearrangement events that occurred after the hybridization but also to have been recent. It must have been one of the first posthybridization events, because the *GDA1-YEF1* breakage that occurred simultaneously with it is the only point of noncollinearity between the A-subgenome and the *Z*. *bailii* genome (apart from sites of interhomeolog recombination or homogenization; Fig 4). It must have been recent because the pseudogene fragments of the broken *MAT* locus have not yet accumulated any other mutations. There are no nucleotide differences in 2,298 bp between the broken *MAT*α_B locus on chromosome 2 and *HML*α_B on chromosome 7. Together, these 2 observations suggest that the interspecies mating that formed *Z*. *parabailii* occurred less than 10^5^ generations or 1,000 years ago [79]. Such a recent origin is consistent with the very low numbers of gene inactivations that have occurred since hybridization, with the fact that most of these are not shared between the 2 sequenced *Z*. *parabailii* strains (only 8 of 27 inactivating mutations are shared; S2 Table), and with the retention of rDNAs from both parents. We expect that, if the *Z*. *parabailii* lineage survives, it will accumulate extensive inactivations and deletions of redundant duplicated genes over the next few million years, as seen in older WGDs.

The net result of the evolutionary changes to the genome is that *Z*. *parabailii* now has 16 chromosomes (all different in structure but containing homeologous regions), 1 active *MAT* locus, 1 active *HO* gene, and 4 silent *HML/HMR* loci. A genome with this structure resembles haploid *S*. *cerevisiae* [1] and is potentially capable of both mating-type switching and mating. We confirmed that both of these processes occur in ATCC60483. *Z*. *parabailii* has a life cycle in which 16-chromosome haploids mate to produce 32-chromosome diploids (Fig 7) that sporulate immediately because the diploid state is unstable; there is no *MAT***a**1 gene, and hence, there is no **a**1-α2 heterodimer. Thus, *Z*. *parabailii* is an allopolyploid that regained fertility by genome doubling after interspecies mating, as a consequence of damage to 1 copy of its *MAT* locus.

Two previous reports that *Z*. *parabailii* strains produce only mitotic spores [31, 80] can be reinterpreted in view of the hybrid nature of the genome. Their experimental data are fully compatible with the meiotic sexual cycle we propose for *Z*. *parabailii*. Rodrigues et al. [80] made a derivative of ISA1307 in which 1 copy of *ACS2* was disrupted by the G418-resistance marker *APT1* and the other copy was not. After sporulation of this strain, all 80 spores they tested were G418 resistant, and all 16 spores from 4 tetrads contained both an intact copy of *ACS2* and an *acs2*::*APT1* disruption, which led Rodrigues et al. [80] to conclude that the spores were made by mitosis. However, this inheritance pattern is exactly the pattern expected if the 2 copies of *ACS2* are homeologs (different Mendelian loci) rather than alleles of a single Mendelian locus and if ISA1307 is a haploid that autodiploidized before it sporulated. Thus, their strain could be described as haploid *acs2_a*::*APT1 ACS2_B*, where the *ACS2_A* and *ACS2_B* loci have independent inheritance (they are on chromosomes 10 and 13 in our genome sequence). Similarly, Mollapour and Piper [31] disrupted 1 of the 2 copies of *YME2* in strain NCYC1427 with a *kanMX4* cassette and found that all the spores produced by this strain retained both an intact *YME2* and *yme2*::*kanMX4*. They concluded that the spores were vegetative, but again, the result is consistent with meiotic spore production if the 2 *YME2* loci have independent inheritance (they are on chromosomes 4 and 6) and if the disruption was made in a haploid strain that autodiploidized before sporulating. The sequence data in [31] allow NCYC1427 to be identified as *Z*. *parabailii* and not *Z*. *bailii* as originally described. Furthermore, in both ISA1307 [80] and NCYC1427 [25, 31], spores are formed in pairs of conjugated cells, similar to Fig 6A. We conclude that ISA1307 and NCYC1427 have sexual cycles identical to the one we describe for ATCC60483.

The evolutionary steps that formed *Z*. *parabailii* by interspecies mating, and restored its fertility by damage to one of its *MAT* loci, are essentially identical to one of the mechanisms (hypothesis B) proposed for the origin of the ancient WGD in the *S*. *cerevisiae* lineage [3, 18–20]. Our study therefore validates genome doubling after *MAT* locus damage as a real evolutionary process that occurs in natural interspecies hybrids, enabling them to resume mating and meiosis. The *Z*. *parabailii* hybridization was very recent, so any period of clonal reproduction that elapsed before fertility was restored must have been short, which is as expected because there is no selection to maintain meiosis genes during clonal growth [18, 20]. The possible role of *MAT***a**1 in the ancient WGD remains unclear. In *Zygosaccharomyces*, the absence of this gene makes zygotes proceed into sporulation. In the ancient WGD, it is likely that a *MAT***a**1 gene was present in the initial zygote, in which case the zygote would have been stable until it sustained *MAT* locus damage, but this is not certain because the ZT parent might have lacked *MAT***a**1. The specific cause of damage to the *MAT* locus in *Z*. *parabailii* was incorrect DNA repair after cleavage by the mating-type switching endonuclease HO. The *HO* gene is present in the ZT clade, but not in the KLE clade [51, 81], and these 2 clades were the 2 parental lineages of the interspecies hybridization that led to the ancient WGD [3]. Species that contain *HO* show evolutionary evidence of repeated deletions of DNA from beside their *MAT* loci, caused by accidents during mating-type switching [51]. Indeed, the disappearance of the *MAT***a**2 gene from Saccharomycetaceae genomes, which occurred at approximately the same time as the WGD, must have been due to some sort of mutational damage to the *MAT* locus. Although HO-mediated damage can only occur in the small clade of yeasts that contain *HO*, other types of mutational damage to 1 copy of *MAT* are a plausible mechanism for fertility restoration in other fungal interspecies hybrids.

## Materials and methods

### Strain and growth media

The strain analyzed here originally came from the collection of Thomassen & Drijver-Verblifa NV in the Netherlands [57, 58] and was called “*Saccharomyces bailii* strain 242” in those studies. It was isolated from citrus concentrate being used as raw material for soft drinks. It was later deposited at the American Type Cultures Collection as ATCC60483. Suh et al. [28] identified it as *Z*. *parabailii* by molecular methods.

### PacBio DNA sequencing, assembly, and annotation

ATCC60483 genomic DNA was prepared using the Blood & Cell Culture DNA Mini Kit (Qiagen), according to the manufacturer’s manual. To prevent fragmentation of the DNA, the sample was not vortexed. The final genomic DNA amount was 15 μg as determined by Qubit Fluorometer (Thermo Scientific). PacBio sequencing was carried out by the Earlham Institute (Norwich, United Kingdom) using 8 SMRT cells, which generated 218x mean coverage for the nuclear scaffolds. We assembled the raw data using the computational facilities at the Irish Centre for High-End Computing (ICHEC), with the HGAP3 protocol of the SMRT Analysis suite version 2.3.0 [82]. We initially obtained 22 nuclear scaffolds, which we reduced to 16 chromosomes by manually identifying overlaps between scaffolds. In parallel, we also obtained 198x Illumina read coverage of the genome (Genome Analyzer IIx; University of Milano-Bicocca, Department of Clinical Medicine), which we assembled separately into contigs that were used to verify the status of rearrangement points and pseudogenes discussed in the text.

The *Z*. *parabailii* chromosomes were annotated using an improved version of our automated YGAP [83], which uses information in the Yeast Gene Order Browser [78] and the Ancestral (pre-WGD) gene order [41] to generate a synteny-based annotation. The automated annotation was curated using transcriptome data from ATCC60483 cultures grown in a bioreactor; Illumina RNA-Seq was generated at Parco Tecnologico Padano (Italy). We made a de novo transcriptome assembly using Trinity [84] and compared the transcripts against YGAP’s gene models using PASA [85] and by manual inspection of spliced mRNA reads.

Chromosomes were numbered 1 to 16 from largest to smallest. Genes were given systematic names by YGAP such as *ZPAR0D01210_B*, where *ZPAR* indicates the species; *0* indicates the genome sequence version; *D* indicates chromosome 4; *01210* is a sequential gene number counter that increments by 10 for each protein-coding gene (genes that were added manually have numbers that end in 5 or other digits); and the suffix *_B* indicates that this gene is assigned to the B-subgenome as described below. NCBI nucleotide sequence database accession numbers are CP019490–CP019505 (nuclear chromosomes), CP019506 (mitochondrial genome), and CP019507 (2-micron plasmid).

The mitochondrial genome of *Z*. *bailii* CLIB213^T^ was not reported with the rest of this strain’s genome [38] and is highly fragmented in the assembly. We identified mitochondrial contigs in the original CLIB213^T^ assembly by BLASTN using the ATCC60483 mtDNA as a query, assembled these contigs into 55 larger contigs using the CAP3 assembler and SSPACE3 [86, 87], and calculated a weighted average nucleotide identity of 96% from nonoverlapping alignments totaling 23,197 bp.

### Gene assignments to the A- and B-subgenomes

We assigned most genes in *Z*. *parabailii* ATCC60483 to either the A-subgenome (highly similar to the *Z*. *bailii* CLIB213^T^ genome) or the B-subgenome (derived from the other parent in the hybridization), using their levels of synonymous nucleotide sequence divergence from CLIB213^T^ genes. For this purpose, we used BLASTP [88] to compare every annotated protein from ATCC60483 to the CLIB213^T^ proteome and designated the best hit as a homolog. The corresponding ATCC60483 and CLIB213^T^ DNA sequence pairs were then aligned using CLUSTALW [89], and their levels of sequence divergence were calculated using the yn00 program from the PAML suite [90]. ATCC60483 genes were assigned to the A-subgenome if the level of synonymous divergence was *K*_S_ ≤ 0.05 and to the B-subgenome if 0.05 < *K*_S_ ≤ 0.25 and given an *_A* or *_B* suffix on the gene name accordingly. Genes for which *K*_S_ > 0.25 or for which no *Z*. *bailii* homolog was identified were given the suffix *_N*. To identify inactivated genes systematically, we searched the annotated A:B gene pairs for cases in which one of the homeologs was less than 90% of the length of the other, and we then examined these cases manually (S1 Table).

Note that our use of the labels “A” and “B” differs from the scheme used by Mira et al. [39] for strain ISA1307. We designated each gene (homeolog) as either “A” or “B” based on its divergence from *Z*. *bailii* CLIB213^T^, with “A” always indicating the *Z*. *bailii*-like homeolog. Some chromosomes therefore contain mixtures of “A” and “B” genes due to posthybridization recombination or homogenization between the 2 subgenomes. In contrast, Mira et al. [39] identified homeologous pairs of scaffolds in their assembly and arbitrarily designated 1 scaffold as “A” and the other as “B” so that each scaffold is homogeneous, but there is no consistent relationship between the “A” and “B” labels and the parent-of-origin of a homeolog in their scheme.

### Tetrad dissection and *MAT* locus PCR amplification

Cells were left for sporulation on malt extract (5%) agar for 5 days. A small loop of cells was washed in sterile distilled water, resuspended in a 1:20 dilution of Zymolyase 100T, and incubated for 10 min at 30°C. The Zymolyase solution was removed by centrifugation, and the pellet resuspended in distilled water (500 μl). A 10-μl drop was placed in the middle of a YPD plate, and dumbbell-shaped asci were dissected using a Singer Sporeplay dissection microscope. The YPD plate was incubated for 2 days at 30°C. Individual spore-derived colonies were used for *MAT* locus genotyping by colony PCR using Q5 polymerase high-fidelity 2x master mix (NEB) and annealing temperature 55°C. Sequences of PCR primers A–F are given in S3 Table. Primers E and F were designed to bind equally to the *HMR* regions of the A- and B-subgenomes. Primers A–D are specific for the A-subgenome.

## Supporting information

S1 Fig Phylogenetic tree of internal transcribed spacer (ITS) regions of rDNA. Chr4 and Chr11 are the ITS sequences from the chromosome 4 and 11 rDNA units in the *Z*. *parabailii* ATCC60483 genome. All other sequences are from Suh *et al*. [28] for strains of *Z*. *parabailii*(Zpar), *Z*. *bailii*(Zbai), and *Z*. *pseudobailii* (Zpse). Letters a-n are ITS variant designations [28]. The tree was constructed by PhyML in the Seaview package using default parameters.
(TIF)

S2 Fig Sequence alignment of the *VPS1-MET14* intergenic regions from *Z*. *rouxii* and *Z*. *parabailii*. The *Z*. *parabailii* regions contain *CEN4* and *CEN11* whereas the *Z*. *rouxii* region is not a centromere. Putative CDE I and CDE III motifs are boxed.
(TIF)

S1 Table *Z*. *parabailii* orthologs of *S*. *cerevisiae* genes involved in mating or meiosis. (XLSX)

S2 Table Homeolog pairs in which one gene is damaged and the other is intact, in ATCC60483. (XLSX)

S3 Table PCR primer sequences used for *MAT* locus genotyping. (DOC)

S1 Data Excel spreadsheet containing, in separate sheets, the underlying numerical data for Fig panels 2A, 2B and 2C. (XLSX)

## Abbreviations

BIR: break-induced replication
chr.: chromosome
ICHEC: Irish Centre for High-End Computing
ITS: internal transcribed spacer
KLE: *Kluyveromyces-Lachancea-Eremothecium*
LOH: loss of heterozygosity
mtDNA: mitochondrial DNA
NHEJ: nonhomologous end joining
ORF: open reading frame
PacBio: Pacific Biosciences
rDNA: ribosomal DNA
RNA-Seq: RNA sequencing
WGD: whole-genome duplication
YGAP: Yeast Genome Annotation Pipeline
ZT: *Zygosaccharomyces-Torulaspora*
