# Supplementary figures and images for: Evolutionary restoration of fertility in an interspecies hybrid yeast, by whole-genome duplication after a failed mating-type switch
Source: PLoS Biol. 2017 May 16;15(5):e2002128. doi: 10.1371/journal.pbio.2002128 (PMC5433688; doi:10.1371/journal.pbio.2002128)

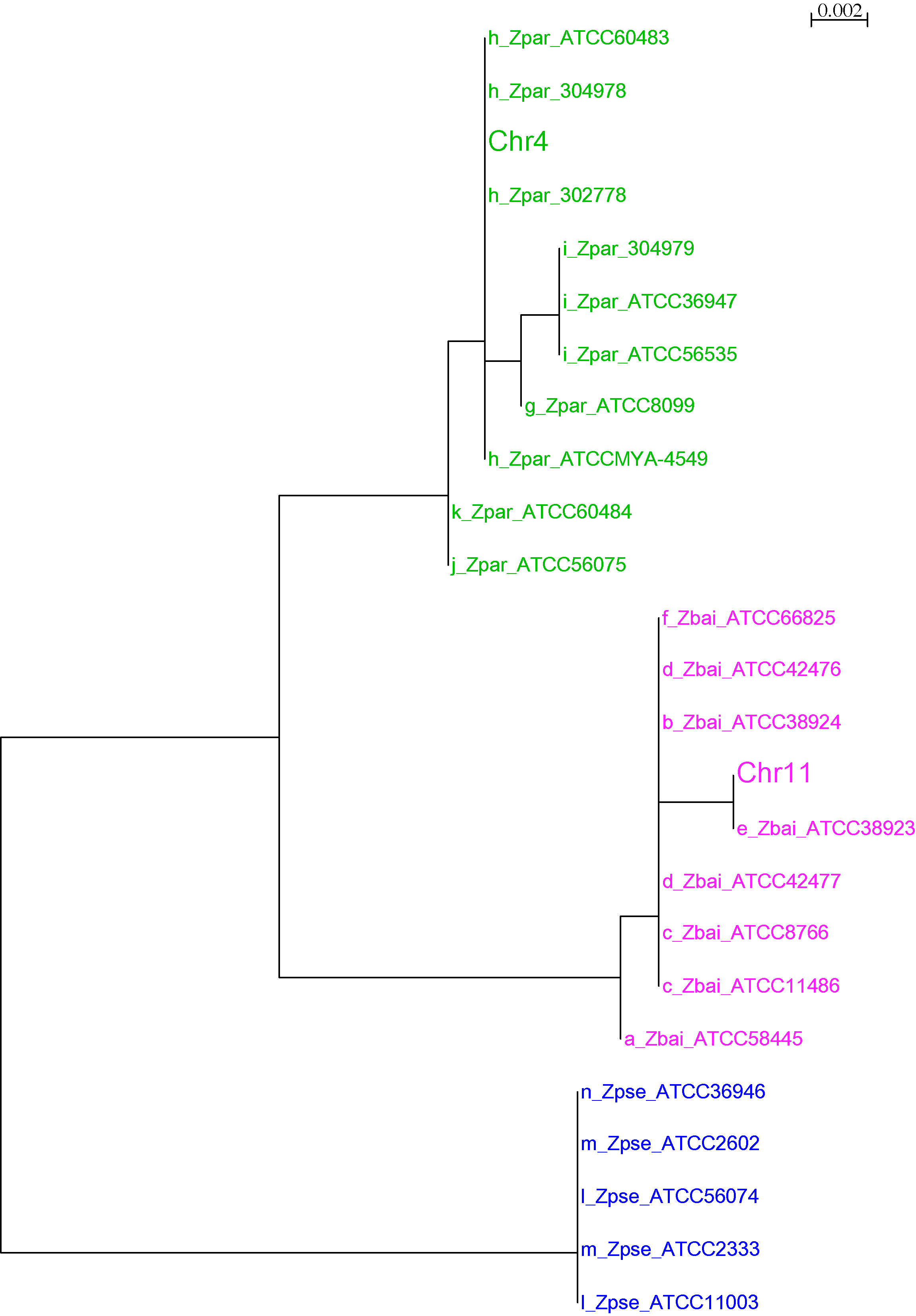

Supplement: S1 Fig — Chr4 and Chr11 are the ITS sequences from the chromosome 4 and 11 rDNA units in the Z. parabailii ATCC60483 genome. All other sequences are from Suh et al. [28] for strains of Z. parabailii(Zpar), Z. bailii(Zbai), and Z. pseudobailii (Zpse). Letters a-n are ITS variant designations [28]. The tree was constructed by PhyML in the Seaview package using default parameters. (TIF) [file pbio.2002128.s001.tif]

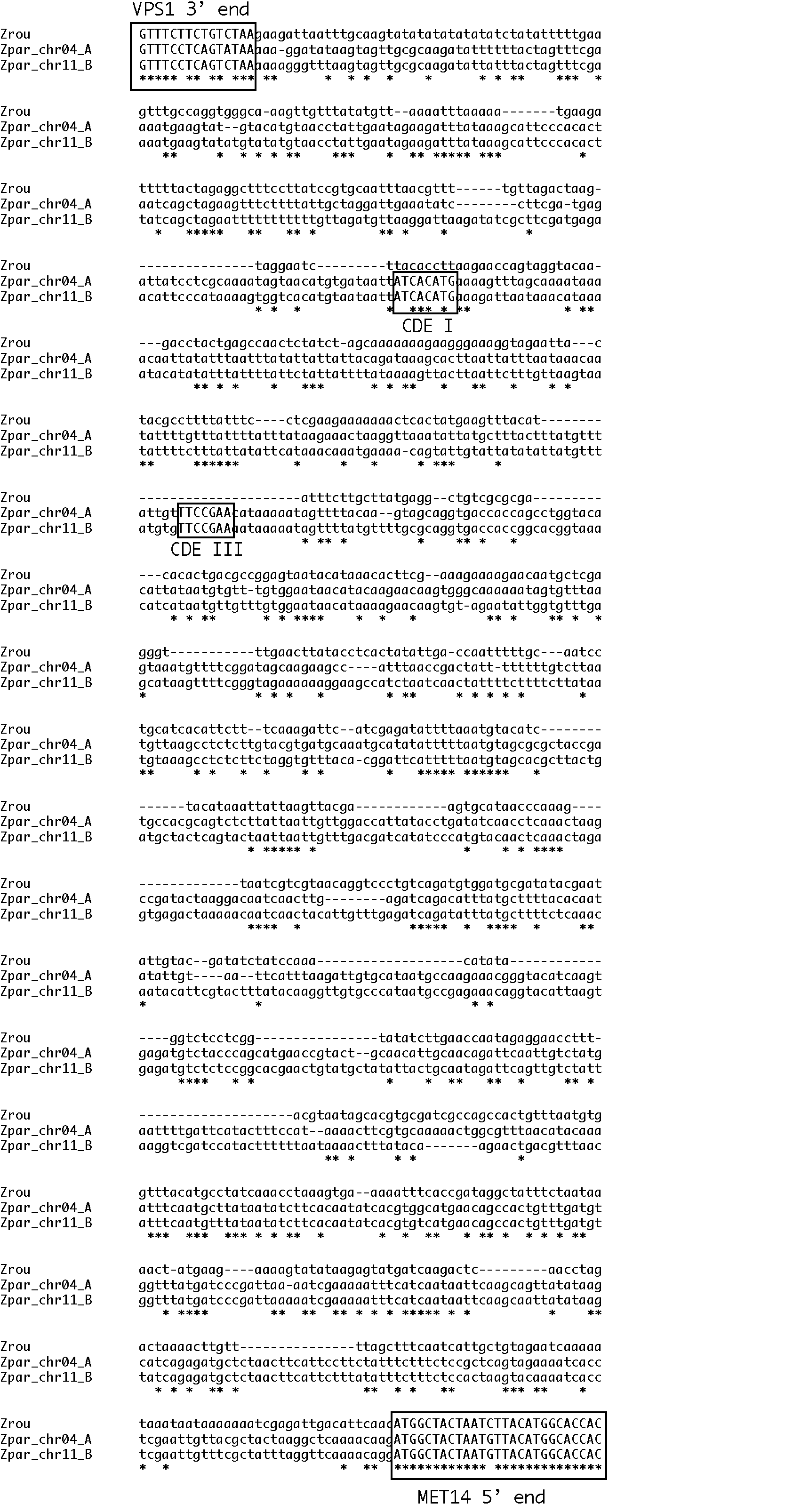

Supplement: S2 Fig — The Z. parabailii regions contain CEN4 and CEN11 whereas the Z. rouxii region is not a centromere. Putative CDE I and CDE III motifs are boxed. (TIF) [file pbio.2002128.s002.tif]
