# Supplementary material for: Evolutionary restoration of fertility in an interspecies hybrid yeast, by whole-genome duplication after a failed mating-type switch
Source: PLoS Biol. 2017 May 16;15(5):e2002128. doi: 10.1371/journal.pbio.2002128 (PMC5433688; doi:10.1371/journal.pbio.2002128)
Supplement: S3 Table — (DOC) [file pbio.2002128.s005.doc]

**S3 Table.** PCR primer sequences used for *MAT* locus genotyping.

| Primer | Sequence (5’ to 3’) | Comment |
| --- | --- | --- |
| A | ttgatggacttacaggatgc | *SLA2*; Specific to A-subgenome |
| B | gggattcaagagtaatcagc | alpha1; Specific to A-subgenome (will also bind HMLalpha_A on chr. 16) |
| C | caaagttaagcaaccaagtgc | alpha2; Specific to A-subgenome (will also bind HMLalpha_A on chr. 16) |
| D | tgatgttgacgatatctgcg | *DIC1*; chrs. 2 and 7 are homogenized A-subgenome |
| E | ggtttggcttttgcgaagg | Binds region identical in *HMR*a2_B (chr. 7) and *HMR*a2_A (chr. 2) |
| F | taagaagaagtcacagaccg | Binds region identical in *HMR*a2_B (chr. 7) and *HMR*a2_A (chr. 2) |

See Figure 5A for approximate locations of binding sites.
